# Gender, aging, poverty and health: Survival strategies of older men and women in Nairobi slums

**DOI:** 10.1016/j.jaging.2007.12.021

**Published:** 2009-12

**Authors:** Netsayi N. Mudege, Alex C. Ezeh

**Affiliations:** African Population and Health Research Centre, P.O. Box 10787, 00100-GPO, Nairobi, Kenya

**Keywords:** Gender, Health, Aging, Poverty

## Abstract

This paper is based on data from focus group discussions and in-depth individual interviews carried out in two slum areas, Korogocho and Viwandani in Nairobi, Kenya. It discusses how the division between domestic sphere and public sphere impacts on survival during, and adaptation to old age. Although this paper adopts some of the tenets of the life course approach, it posits that women's participation in the domestic sphere may sometimes give them a ‘gender advantage’ over men in terms of health and adaptation to old age. The paper also discusses the impact of gender roles on the cultivation of social networks and how these networks in turn impact on health and social adjustment as people grow older. It investigates how older people are adjusting and coping with the new challenges they face as a result of high morbidity and mortality among adults in the reproductive age groups.

## Introduction

Aging discussions generally focus on the disadvantages people face as they grow older. These disadvantages can range from systematic stereotyping and discrimination of older people which, in the literature, is commonly referred to as ageism to chronic illnesses that increase in prevalence with age. For instance, Butler (1983:360) notes that only 14% of the over 65s are free from chronic diseases. Studies on African ageing postulate that there are higher incidents of sickness and ill-health among older people compared to population averages (Faye, 2007; Barrientos, Gorman, & Heslop, 2003). Studies on diseases of old age abound in literature as people seek to improve the quality of life of older people through better health care and access to amenities (Hood, 2003; HelpAge International-Africa, 2001; Bennet, 2001; Cleveland, 1976).

Another widely used approach, particularly in the social sciences, has been the life course approach (Knodel & Ofstedal, 2003:679). This approach investigates what individuals go through in their lives that can disadvantage or advantage them as they get older. This approach has looked at things such as poverty or gender discrimination and how these can predispose individuals to certain disadvantages as they grow older. Poverty experienced earlier in life can predispose individuals to certain illnesses in their old age. Sometimes exposure to toxic substances and environments earlier during working lives could predispose older people to diseases such as cancer or blindness. For instance, in the two study communities (Korogocho and Viwandani) a higher percentage of men suffer from respiratory illnesses. Ezeh, Chepngeno, Kasiira, and Woubalem (2006) notes that 22.5% of men compared to 15.2% of women suffered from respiratory illnesses. Also earlier persistent poverty can make people vulnerable to diseases like sugar diabetes in older ages as a result of poor diets. WHO (2005) points out that, ‘there is now extensive evidence from many countries that conditions before birth and in early childhood influence health in adult life. For example, low birth weight is now known to be associated with increased rates of high blood pressure, heart disease, stroke and diabetes’. It has also been noted that although diabetes affect people regardless of their socio-economic status, people in poor areas are affected more than those in wealthier settings (Peláez & Vega, 2006).

The life course approach also postulates that discrimination, for example, against women and other marginalized groups can result in them experiencing poverty in old age if they were excluded from high paying jobs earlier in their lives. WHO (2005), for example, linked pathological aging to poverty; it noted that ‘Chronic diseases and poverty are interconnected in a vicious circle. At the same time, poverty and worsening of already existing poverty are caused by chronic diseases. The poor are more vulnerable for several reasons, including greater exposure to risks and decreased access to health services’. However, Knodel and Ofstedal (2003) have criticized the life course approach for always focusing on female disadvantage. In a 2003 paper, they pose the question ‘where are the men?’ They argued that older men's problems are being under theorized as a result of an uncritical bias towards women as a permanent minority. In the East Africa region, Knodel and Ofstedal (2003) observed that 46% of the population above 60 years is male and women's advantage in life expectancy (also known as the gender advantage) at age 60 is 1.5 years. Knodel and Ofstedal (2003:682–683) noted that ‘men represent a declining share of successive older age groups among the elderly’. For instance, whilst world statistics indicate that men comprise nearly half of the older persons in the 60–64 year age group, they make up only two fifths of the population of older people who are 80 years and above (UN, 2002, cited in Knodel & Ofstedal, 2003). This paper does not assume a permanent male advantage or a permanent female disadvantage, but seeks to investigate how the perceived female disadvantage of relegation into the domestic sphere can be an advantage as people grow older.

This paper investigates how the domestic and public sphere separation impacts on health and survival strategies and abilities during old age. It discusses how the division between the domestic sphere (characterized as a predominantly female domain) and the public sphere (characterized as the male domain) impacts on survival during and adaptation to old age. Although this paper adopts some of the tenets of the life course approach, it posits that, sometimes women's participation in the domestic sphere can give them a ‘gender advantage’ over men in terms of health during old age and adaptation to old age in general. It discusses the impact of earlier gender roles on the cultivation of social networks and how these networks, in turn, impact on health as well as social adjustment in old age. The paper also examines on how older people are adjusting to the new demands they experience as a result of high morbidity and mortality rates among young adults in the reproductive age groups occasioned by the HIV/AIDS pandemic. Available literature on Africa has been unanimous that the scourge of HIV/AIDS coupled with rapid urbanization and increasing poverty has made African families less able to offer support to older people (Lloyd-Sherlock, 2000; Aboderin, 2004a,b). Lastly the paper examines the links between health and poverty among older people and offer policy prescriptions for meeting the wellbeing of older people.

## Methodology

This paper is based on qualitative data collected from Korogocho and Viwandani slum areas in Nairobi, Kenya. The data comes from an Aging study nested onto the Nairobi Urban Health and Demographic Surveillance System (NUHDSS), a longitudinal study implemented by the African Population and Health Research Center (APHRC) in the two slum communities since 2000. The NUHDSS involves regular visits to every household once every four months and covers about 60,000 people in some 23,000 households. Using the NUHDSS as a sampling frame, APHRC designed the nested study to qualitatively assess the perceptions and roles older people play in the slum communities and their coping strategies. The Aging study focused on issues such as how people define an older person and the roles and responsibilities expected of older persons. It also investigated the support networks of older people and sought to understand issues of discrimination and abuse of the elderly. Needs of the elderly, especially health needs and how poverty impacts on their health and well being as well as the impact of HIV/AIDS on the elderly were examined.

The Aging study had two components: focus group discussions and individual in-depth interviews. The focus groups were more encompassing in terms of age. The sample covered community members aged 15 years and older differentiated by age and gender with a total of 13 FGDs in each community. Table 1 indicates the composition of the focus groups.

The field supervisor who is normally based in the field recruited participants for the focus group discussions by announcing and explaining the aims of the research to the community at community forums as well as making follow ups to people's homes to recruit and seek permission.

The second part of the study consisted of older respondents who were selected for individual in-depth interviews (IDIs). The sample was a stratified non-random sample that was designed to capture a wide variety of characteristics among the older people. The Demographic Surveillance System (DSS) data was used to identify the respondents. Thirty-two IDIs were conducted. The sample of individuals was drawn to cover the following categories of older people: those dealing with recent death of an adult child, looking after orphans, disabled old people, those who lived alone. The sample also had to balance for gender of the respondents. However, some of these categories overlapped. For instance, an older person dealing with a recent death of an adult child could also be caring for orphans, or a disabled person could be living alone. Out of the six men living alone, three had wives living in the rural areas. On the interview of older people looking after orphans where the household consisted of husband and wife, we normally interviewed the head of the household who was usually male unless the male refused to be interviewed. For instance, in Korogocho, one male refused to be interviewed pointing out that it was the wife who looked after orphans therefore should be interviewed. He claimed not to know a lot on orphan care. Table 2 illustrates the composition of people involved in the individual interviews.

All focus group discussions were conducted in Swahili by native speakers. Twenty-one in-depth interviews were carried out in Swahili while the remaining eleven were conducted in one of the following languages Kikuyu, Luo and Luhya. The language of use was determined by the preferences of the interviewee. All focus group discussion and in-depth interviews were tape recorded; the tapes were then transcribed and translated into English. The interviews were then coded using Nudist, a coding, software used in the analysis of qualitative data.

Some reference is also made to the Information for Development (IFD) study also nested on the NUHDSS and carried out in the same year as the Aging study. Some of the notions of gender role patterns and socialization in the community discussed in this paper are generated from the IFD study which focused on the roles boys and girls play within their families and communities and how these may affect their schooling outcomes. The implications of gendered role patterns on adjustments at old age are then examined using the aging study.

### Definitions

Old age officially has often been pegged at retirement ages which may vary from country to country. For most countries, the retirement age is between 60 and 65. Many academic papers arbitrarily use the 60–65 age group as a cut off point, for defining older people. ‘The United Nations identifies….populations who have reached the age of 60 years as “older persons”’ (Huber, 2005:2). The retirement age in Kenya is 55. Using the chronological age of 60 years, Huber (2005:3) pointed out that between 2006 and 2050 the proportion of the aged population in sub-Saharan Africa is expected to double from 5% to 10% being the highest increase in older people in the different regions of the world. Some studies in Africa use age 50 as the arbitrary identifier of an older person (see McIntyre, 2004).

Despite the wide meanings and perception attached to the concept of being an older person in the study communities (see Ezeh et al., 2006), this paper regards people aged 50 and over as elderly. This is because most of the things that affect the elderly such as physical impairments and declining health, in the slums start manifesting at relatively younger ages. It also uses functional and social markers to identify old age (Glascock & Feinman, 1980 for alternative ways of defining old age without limiting oneself to the arbitrary official retirement age).

The domestic sphere refers to anything that has to do with the family and home. Tasks that are associated with the domestic sphere include looking after children, cooking, washing clothes and looking after the sick and elderly. The domestic sphere is solely concerned with the social reproduction of individuals within the family. The public sphere normally has to do with the creation of material and non material values that can be exchanged because of their market value whilst domestic sphere activities are without value in market terms (Azmon, 1981:553). Domestic sphere activities are often imbued with symbolic values.

The domestic sphere includes all activities organized immediately around the mother or mothers and their children. The public sphere covers all the activities outside the bounds of these relationships. The women are identified with the domestic sphere, while the men are identified with the public sphere (Azmon, 1981:550).

The public sphere is associated with the market and politics, and, domesticity draws boundaries between the home and the world of men (Kaplan, 1998:586; Ryan, 1998).

According to the 1997 Kenyan Welfare Monitoring Survey III, in Nairobi 42.1% of men aged 15 to 65 compared to 17.8% of females in the same age group are employed in the public and formal private sectors (see Atieno & Teal, 2006). Table 3 shows the working status of older men and women in Korogocho and Viwandani.

Although women working in formal employment can be said to be working in the public sphere, their traditional preexisting workloads within the domestic sphere may not diminish hence the continued reference to the domestic sphere as predominantly female. Alessandrini (2003:3) refers to this as the double shift, since the women have to work at their jobs and also need to accomplish their traditionally defined feminine roles at home. Discussing the double shift, Baxter and Western, (2001:81) wrote:

Most men tend to be in full time employment and to do only small and unchanging amounts of unpaid work regardless of institutional arrangements, life course stage, or socio-economic characteristics….While men are able to keep the boundaries between work and home relatively distinct, women experience much more overlap.

Although women may take up employment in the formal sector and occupy political office (public realm), they are still more associated and committed to the home than are men. This applies to Korogocho and Viwandani where some of the women work outside the home in both the formal and informal sector but still shoulder the burden of their domestic responsibilities.

### Study context

Korogocho was officially settled in December 1978 and covers an area of about 49.2 ha. It is located about 12 km from Nairobi city center. To the east and south east of the slum settlement is the Nairobi Refuse Dump site where some of Korogocho residents make a living by scavenging for food or things like scrap metal for sale. Korogocho, developed on land originally owned by an individual called Baba Dogo and what was left by the City Council as a reserve land on the banks of the Nairobi and Gitathuru rivers. Houses in Korogocho are mostly made of mud or timber with roofing composed of tin waste. Most of the residents of Korogocho are either uneducated or dropped out of school at primary level; only 19% of men and 12% of women attained a secondary education.

**Fig. 1:**
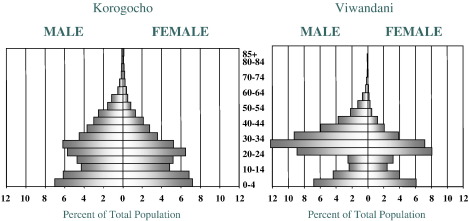
Population pyramid of Korogocho and Viwandani.

On the other hand, Viwandani was officially recognized as a settlement in 1973, covers an area of about 3 km length, and 1 km width. It is located about 7 km from Nairobi city center. It developed on land that was left by the City Council as reserve land on the banks of the Nairobi river. To the North of the settlement are the industries where many of Viwandani residents earn a living by working in the factories. The presence of industries explains why there are more men in the economically active group in Viwandani compared to men in the same group in Korogocho. However, no one knows precisely when the original settlers moved into Korogocho and Viwandani. Fig. 1 indicates the population pyramid of both Korogocho and Viwandani.

For most people, the slum is what they regard as home as they have no where else to go. One of the most frequently cited reason for not moving away from the slum at old age was the lack of land in rural areas. A growing number of young people have also been born in the slums, often out of wedlock and the slum is the only place they know as home. An 83 year old man in Viwandani taking care of orphans had this to say,

Res^1^: I have lived in this village for a long time because those are days there were no structures here. There were hyenas….There were hyenas, and other the wild beast.

Int: Which year was this?

Res: It is like I was born here.

Int: So you don't know which years was this?

Res: I cannot remember. It is like I was born here…

Int: Why have you lived in this community for all these years?

Res: It is because I don't know anyplace that I can go. I don't have a farm. Not even a quarter.

Thus, although for a large number of old people the slum is their home, a few others still maintain links to their rural home. For example, a 72 year old man living alone, had moved his wife to the rural areas after they had been left with many orphans to look after. However, for the majority of young people born in the slums, the slum is home, it is what they have always known.

When asked when she had arrived in Korogocho, an elderly woman who did not know her age said,

Res: I cannot remember.

Int: What time did you come here?

Res: After the Mau Mau war, when Korogocho was being constructed but I cannot remember when.

Int: Do you intend to go back to the rural area?

Res: Where do I go? I do not have land there. Where do I go back to?

Int: What about your home?

Res: My father did not give me land.

Int: I am talking about your husband's home, the one you said died?

Res: After he died his brothers sold all the land he had left. So I went back to Murang'a where I was born but also there I was not given land by my father.

Although the dates given by the respondents on when they first settled in Korogocho contradicts the official dates, these personal histories of respondents indicate that most of the original settlers have spent most of their life in slums and regard it as their home. In the case of the above woman, she claims to have settled in the slum after the Mau Mau war which ended in 1960. She could have settled in Korogocho in the early 1960s, and the slum has been her home ever since.

### Socialisation, work and gender

Other qualitative studies conducted by the African and Population and Health Research Center (APHRC) in the same study communities have consistently shown that girls do most of the domestic chores as compared to boys. For example, a 2004 study nested on the NUHDSS investigated factors that led students to drop out from school. One of the key factors affecting girls emerged to be their heavy domestic work burdens. A boy in the 12–14 year old focus group discussion summarized the prevailing notions of gender roles and how boys and girls, even in urban informal, settings are being socialized into these roles.

Some people think duties like washing faeces and plates are duties for the ladies and not boys… The mothers say that all of the house duties are for the ladies, like washing plates, clothes, cleaning the house and staying with the baby. The boys' work is only fetching water — easy job.

This notion of gendered domestic roles was a common thread in most of the interviews in that study with girls doing most of the domestic tasks.

Across ethnic groups, gender, socio-economic and age groupings, girls are regarded as primarily responsible for domestic chores, the only exceptions appear to be when there is no girl in the home, then the male children also shoulder the burden of domestic work.

Studies elsewhere in Africa also observed similar findings that girls shoulder the burden of domestic work. For instance, ILO (1999) pointed out that although in African official statistics, boy outnumber girls in child labour, it is because unpaid domestic work such as cooking and looking after the sick and disabled is not included in the statistics. Boys generally take up jobs for pay away from the confines of the home. For example, young boys can work at the market, become car washers, sell scrap metal or worked as Mukokoteni (i.e. carrying people's luggage for pay using hand made pull carts).

In interviews and focus group discussions, it emerged that boys are the only ones who could pick scrap metal and wash cars. The most commonly cited jobs for girls were being a maid or prostitution. Girls could use the skills they gained from performing domestic task within the home to earn a living when they became maids. According to girls aged 12–14 in Viwandani, the role of boys in working outside the home is very clear. Their group discussion proceeded as follows:

Res5: Most of such children drop out of school, both boys and girls so that they can go and fend for themselves. For a boy, he goes and collects scrap metals or woods and sells to potential buyers. The money they get, they use to buy food for their families. Some of them go to town and become street children who then collect leftovers and take to their siblings.

Mod: Those who do such, are they boys or girls?

All: boys.

Thus girls are normally confined to the domestic sphere. This pattern of gendered public and private spheres is not unique to Kenya. Similar findings have been observed in other African societies. For example in Zimbabwe, Cheater and Gaidzanwa (1990:191) point out that in Shona societies, ‘traditions of male mobility contrast sharply with female immobility’. Women who were highly mobile and ventured out of the bounds of their immediate residential neighbourhoods were often labelled as prostitutes. Such negative association between female mobility and prostitution sometimes reinforce domesticity where girls and women are concerned. This divide may be further widened in some societies by religious prescriptions such as purdah among the Moslems where women's work outside the home may be proscribed.

### The link with adult roles

The roles that these children played and the jobs that they did seemed to be mirrored in the roles that adult men and women play in the slum communities. When asked what older men did to earn a living, a woman in a focus group discussion for 50–59 year old females in Korogocho said:

The elderly men go to the construction sites, dumping sites to collect used bottles and plastics and take them to sell to industries and are paid, while others may go to farms in Kasarani or Kariobangi south. Everyone knows where they go to look for their livelihood.

On the other hand, older women were more likely to be involved in petty trading which could be done within the community.

Some respondents in the focus group discussions pointed out the fact that women aged faster as compared to men. According to older women in a focus group discussion in Korogocho:

Res1: There are situations whereby one may have married and has children who still want to depend on their parents. This becomes a burden for the mothers as they cannot send away their children. This is what causes the women to age faster.

Res3: Some also take care of grandchildren whom they have to feed and clothe.

Many discussants and interviewees blamed the huge amount of domestic work that women did within their homes for the perceived faster aging of women compared to men.

## Old age and the myth of the ‘role-less role’

Enerst Burgess (cited in Cole, 1983:35) wrote that when people grow older, they get into a role-less role where they do not have anything to contribute anymore. This was particularly true for retired males who could no longer act as the provider and protector but instead have to be protected and provided for by their offspring. This, according to Burgess, resulted in alienation for older people and a feeling of helplessness, and unworthiness which can be psychologically damaging. For example, being sent to an old people's home can be disconcerting for the older people. This is particularly true of well developed countries with well advanced social welfare systems and where people's identities are closely linked with their jobs.

Contrary to Burgess' assertion that when people get older they gradually enter the role-less role, the opposite is often true for developing countries ravaged by illness and high mortality and morbidity among young adults. Older people in sub-Saharan Africa have a lot of burdens to shoulder. As indicated above in the excerpt of a 70 year old man who was still responsible for paying school fees for his grandchildren. Instead of the old relinquishing most of the tasks they did before getting old, they tend to take up more of the same tasks they shouldered as young adults. With the advent of HIV/AIDS, some older people may find themselves looking after large families especially if more than one of their children dies of the pandemic. This is a growing trend in countries ravaged by HIV/AIDS particularly in sub Saharan Africa. For instance, 61% of double orphaned children and single orphans not living with surviving parents in Namibia, South Africa and Zimbabwe and over 50% in Botswana, Malawi and Tanzania are living with their grandparents. About 30% of all households in sub-Saharan Africa are headed by an older person (Monasch & Clark, 2004). Fifty-six percent of care givers for orphans and vulnerable children in Namibia are more than 60 years old (Monasch & Clark, 2004).

When women in a 50–59 year age group in Korogocho were asked to explain how older people were coping with the problem of orphans this is what they said:

Res1: Someone like me I have an orphan whose parent died of AIDS, it has been very difficult for me as I have to see to it that he goes to school, has uniform, clothes and at least one meal a day. At times we go without food when I don't get any work to do.

Res6: I have three orphans whom I look after and it is very difficult. I look for food and casual work to be able to help these grandchildren of mine.

Res8: I know of a shosho (grandmother) whose two adult children died of AIDS and she has now ten orphans. She sometimes get support from her other children but it is never enough to feed all these grandchildren.

In-terms of access to formal jobs, older people may generally be relegated to a role-less role at a time they have to look after increasingly large families. Instead of retiring into the ideal graceful old age where they are looked after by their children and grandchildren, older people have had to work to support their growing number of dependents.

In addition, these older people will have to take on these roles at a time when their health is also declining because of old age. In a focus group discussion of old men of 60 years and above in Korogocho, it was observed that older people suffer from a variety of illnesses.

Mod: What are the health problems of the elderly here?

Res4: If I look around I see Tuberculosis, Asthma, whooping cough and diabetes.

Res1/8: We affirm what he has said. We also have backache, limb pains and joint pains.

Most older people in Korogocho and Viwandani pointed out that they had problems with chronic pain in their legs and backs that restricted their movement. In urban informal settlements, this is occurring with little social support from the extended family or from private and public agencies. When asked to state who looked after orphans in their community, some men in a focus group discussion of men who were 60 years and above in Korogocho stated that:

Res5: Sometimes some of us, Luos and Luhyas, after parents die we take the orphans home in the rural areas and after that they stay with their grandparents. There is nothing else we can do for the orphans.

Res8: Us, Kambas, if the parents die we take the orphans home to stay with their grandparents.

Mod: Let us say it is you, what could happen?

Res8: The children could be taken back home to my parents to take care of them.

Mod: What if your parents stay here?

Res8: They would have to go back home because here there is congestion and life in the rural areas is not congested and it is better.

Added to their increased burdens, older people are discriminated against in terms of accessing jobs, and lack incomes to shoulder the new responsibilities of looking after young grandchildren left behind by deceased children. Since men are used to formal jobs or to jobs outside the home, they may find it difficult to subsist in conditions where there are no formal jobs and where they are regarded as generally unemployable because of their advanced age. In the group discussions, it emerged that some older men in the slum communities could not work anymore because of old age and those who wanted to work were normally not hired in the higher paying construction jobs or other jobs in the industries. Instead some of them stayed at home, went into the car washing business and, if they were not too old, could be employed as security guards.

## The domestic and the public divide

The private and public divide, where men are supposed to work in formal employment and women run the affairs of the home or take up informal employment has impacts on adjustments during old age. The distinction between the public and private spheres ‘has served to confine women to typically female spheres of activity like housework, reproduction, nurturance, and care for the young and sick, and the elderly’ (Benhabib, 1998). Women find themselves in the domestic realm where they are expected to nurture the family whilst the husbands go out of the domestic sphere to the public sphere where they compete on the market and in politics. Men can participate in local level politics as a way of accessing resources. For example, they can take part in local council politics as a preparation for longer political engagement. On the other hand, the informal sector provides women with a range of activities that can be combined with their domestic responsibilities (Atieno & Teal, 2006). This point is clearly evident in the following quotation from an individual in depth interview with an older woman in Viwandani:

Before I used to do business when my children were small. I used to work with Cocoa company and when I got a baby I lacked someone to take care of the baby. Taking care of the baby meant abandoning my job. I therefore started business. I would tie the baby on my back and go to the city council market, Marikiti, and buy green grocery to sell here.

Women in the slums normally take up tasks such as petty trade or part time work such as washing people's clothes, work that enables them to stay closer to their homes while still earning some income. For instance, the NUHDSS data show that 73% of women in Korogocho and Viwandani are engaged in petty trading compared to 50.2% of men. On the other hand, 17.4% of women in Viwandani compared to 39% of men are employed in the formal sector (see Table 3). In focus group discussions and individual in-depth interviews, it emerged that most of the female petty traders were involved in selling vegetables and cooked food — activities that they could carry out at home or within the same community. Although some women in the informal sector sometimes work for cash payments, their work is still domestic since they perform typical domestic chores such as cooking, ironing, washing and cleaning for households or for individuals within the slum as well as neighbouring non-slum communities. On the other hand, men take up formal employment or high paying informal jobs that sometimes take them away from home. These jobs usually come with relatively higher returns and sometimes with a stable salary at the end of the month and a possibility of pension towards the end of their working life.

Given that men are used to formal jobs or to jobs outside the home, they may find it difficult to subsist in conditions where there are no formal jobs and where they become generally unemployable because of their advanced age. Evidence from Korogocho and Viwandani suggests that older men sit helplessly in their homes everyday for they cannot find anything to do to raise an income to look after themselves and their families. When asked what he did for a living, a 72 year old man living alone in Viwandani had this to say:

Before, I was selling timber for construction, but the government stopped that business. Now there is nothing to do because I cannot go to the forest. Now I am doing nothing until the day the government will allow us to go back and fetch timber. …When I get 100 shillings I go to my children in Githunguri and I am given cabbage and bananas to come with.

Another older man aged 74 from Korogocho said,

Before, I was not just sitting around doing nothing, I had a job. I had business and hotels, I had a shop, Kiosk and such things. Now with all the money gone, all these things are gone too. All these are gone because with sickness (of his wife and some children), went the money. All my children had children and I stayed with them and fed them. Now I cannot feed them. Now I just wait for money from this house. I cannot go looking for a job. No one will employ me just to sit around. Who can employ anyone just to sit around?

These examples show that sometimes old men find it difficult to adjust to changed circumstances. The general despondency among men in the qualitative Aging study contradicts quantitative statistics that indicate high levels of economic activity among older men. In focus group discussions, regardless of age and gender, older men were generally regarded as and even regarded themselves as unemployed and dependent on others. It is highly likely that although in the quantitative data a higher proportion of men were reported to be working, some of them may be doing jobs for which they are not paid or jobs where the pay is not enough to meet their basic needs. It could also be that for the proportions not engaged in economic activities, the older men may be much more dependent on others for support and assistance whereas older women may be found as actually supporting their households through care of children and assistance with other household chores.

There is anecdotal evidence that older men experience some disadvantages as they grow older because of roles played earlier in life. The following discussion with a 70-year old man in Korogocho summarizes how gender role in earlier life may disadvantage older men in adjusting to life in old age. The 70 year old man had relocated his wife to the rural area to take care of orphaned grandchildren whilst he worked in Nairobi and sent money home.

Int: What kind of work do you do?

Res: I was a watchman at a place where I have finished six months without pay. So I quit and found a different watchman job the other day with another company. Now I am still with the new company working as a watchman but since I joined the company to date I have not been paid yet…

Int: Who does household chores for you?

Res: Since the house is small and I live alone I do not make it dirty. My sister in law sometimes comes to clean it for me. Like today my sister in law came, lit the charcoal burner and she is the one who put for me the food that is cooking on the fire…. I keep getting worried about my people in the rural area are managing since I cannot be able to send them money. Recently a letter I showed you came from my people in the rural area that school fees are needed. My wife says there are problems. The house I had built at home is falling and they are hungry, they need money. They want me to send them at least 500 shillings.

Not only is the man interviewed above engaged in jobs seen by younger people as unattractive which often pays little or nothing, he appears unable to take care of basic needs including depending on female relatives to assist him with cooking and cleaning.

The notion of gender advantage in adaptation to old age is aptly illustrated in the following perceptions of men and women, old and young, in the two communities regarding how older women and men cope with old age. In general, older men appear ill-equipped for the inescapable realities of growing old in urban informal settlements where traditional extended family ties that tended to mitigate such risks are generally weak or non-existent. For older women, however, their life long engagement in the domestic sphere appears to confer unique advantages to coping with life in older ages in the urban environment.

Older men are generally perceived as idle and weak and incapable of caring for themselves. In a focus group discussion with community leaders, they pointed out that older women were more resourceful whilst men were always worrying.

Mod: Is there a difference in what men and women do to support themselves?

Res6: Elderly men are normally weaker than the women.

Mod: What do you mean by weaker?

Res6: Men just normally sit and idle around while women will go out to look for food.

Res5: You will also find that it is the women who come together to form groups that can assist them in times of need.

Res7: Women are generally very cooperative.

Mod: Is there any other difference in the roles or responsibilities?

Res8: Elderly men do not live for so long. Because they worry a lot they die before their age.

Res9: And they die fast because of lack of food, there is no strength to look for this food and generally no one cares. (Male Community Leaders in Korogocho)

The high prevalence of HIV in most parts of sub-Saharan Africa has affected many young people in the reproductive age groups. UNAIDS (2004) estimate the HIV/AIDS rate in Kenya at 6.1%. According to the report, in 2004 alone, a total of 1.2 million Kenyans had the HIV/AIDS virus, 150,000 people died of AIDS in the same year and there were a total of 650,000 orphans. According to the HIV Insite (2007), in 2005 in Kenya the number of orphans increased to 1,100,000. Out of the total population with HIV/AIDS, 740,000 were women between the ages of 15–49 (http://hivinsite.ucsf.edu ). This indicates that a high number of young people in the reproductive age group die or are incapacitated by sickness as a result of high HIV infection, the older people have to take on most of the roles of their young adult children. A male discussant in the 25–29 year age focus group discussion in Korogocho maintained that:

If you go to Kenyatta hospital, most sick people will be from Dandora and Korogocho, and they are suffering from AIDS.

Indeed, an ongoing sero-prevalence study being conducted by the APHRC researchers put the HIV prevalence rate in Korogocho at about three times the national average. Where the remaining adult is an older man with young children or, in cases where the older man is left alone after family members move away or die, the older male will face difficulties because he is not versed with the mundane domestic chores which are critical for survival.

Although older women find it very stressful to adapt to these added responsibilities, their position may be better compared to that of older men as there is not as much role disruption or role incompatibility for them. A participant in the 50–59 year old female focus group discussion in Korogocho said in relation to the differences between men and women:

Res3: Some (older women) also take care of grandchildren whom they have to feed and clothe.

Res1: At times men run away from these responsibilities when problems become too many. They move away and look for other places to stay. In fact I have a neighbour who has been locked out for three weeks now and the husband has gone missing.

This is what some men in the 60 years and above focus group discussion in Viwandani said regarding the differences between older men and women:

Res2: Here it is the women who support us. We would appreciate if you people can give us programs.

Res4: Women here do more roles. Women here who work are in these small businesses. Men look for jobs like watchmen, builders but their age does not allow them to do this kind of work. Most are watchmen but the wages they receive are what they use to buy bottles of beer in the restaurant. The government should do something about this.

Women found it to be their role to look after these orphans and were more often than not resigned to their fate. In taking on these responsibilities, women are doing what they have always done: looking after their families. Indications from qualitative research are that older women perhaps cope much better than older men with respect to these new responsibilities as well as with old age more generally. However, this does not make it less stressful because these are responsibilities that older women are taking up when they do not have the economic and physical strength to do so and when, as one of them observed, ‘our children are supposed to be looking after us’.

It is important to note that traditionally, older people have relied on their children for support in old age. In the absence of social support systems for older people and private investment opportunities, most adults in Africa see their children as the only guarantee to a descent life in old age. However, the full import of the current transition whereby older people lose their main source of support while at the same time taking on added responsibility of caring for their orphaned grandchildren is not fully known. More importantly, how these transitions are impacting on the lives of older people in urban informal settlements already ravaged by growing urban poverty and lacking the support of the extended family deserves urgent attention.

Older men left with the burden of caring for orphaned grandchildren could marry younger women to assist them with such responsibilities. Older women rarely get remarried. For some older men, however, the sheer pressure of having to take on roles they have never considered male roles and losing out on opportunities to play the provider role, could be very damaging to them psychologically. Although the economic, social, and physical costs of taking over these responsibilities may be similar for both older males and females, the stress can sometimes emanate from different angles.

Although life expectancy at age 60 takes into account a number of factors before age 60 that condition survival after age 60, high stress levels caused by too much worrying in men over the age of 60 can be a contributory factor to early death among older men compared to women. A 61 year old man had this to say:

I worry too much. Wherever I am I worry. I wonder about life too much. I think it is because of many worries that I even got TB (Very sad as he speaks)…. I have no bearing in life. I am like a lost man. This forces me to think so much.

This stress — which might be associated with early deaths among older men — in the case of the two slums may be caused by their lack of adjustment to the increasing responsibilities they face. This lack of adjustment to life for older men can also be evidenced by the propensity of older men to run away when there are problems. Although we are not sure how widespread this phenomenon is it was frequently referred to by participants in both the FGDs and IFDs. This is because during most of their working lives, men work outside the home and rarely concern themselves with the day to day running of their households. All older men in the individual in-depth interviews had worked outside the home either at construction sites or timber companies and one had worked as a farm labourer in Uganda. As noted by Mukui (2005:5).

The economic role of the man in a household may be more important when he lives away from the home (especially if working), the denial of emotional support and conjugal rights notwithstanding.

When faced with these new demands and added responsibilities, older men become stressed and as some respondents in the discussions pointed out, older men die earlier than older women and some resort to alcohol or run away from home. Data suggests that older men are failing to handle these stresses well as compared to older women. The following group discussion with 25–49 year old men in Viwandani underscores the difficulty older men face in adjusting to the realities of growing older in the slums.

Mod: Are you saying that there are no many elderly men in this community?

Res5: They are very few if any.

Mod: What do they do to support themselves?

Res5: Truly speaking, some of these elderly men resort to illicit brew when the going gets tough.

Mod: Where do they get the money to drink illicit brew?

Res5: They sometimes get casual jobs but when the problems get too much, even the little they get from casual jobs, they drink it.

In turn, alcoholism can expose men to other chronic and potentially life threatening illnesses. The explanation for ill adjustment by men might lie in the fact that, as they become older, men may find themselves being relegated to the domestic sphere, a sphere they were not socialized into and therefore find it overwhelming to cope with. The lack of coping mechanism to deal with growing old in poor settings, may explain early death and poor health outcomes for older men as compared to older women.

## Social networks and the domestic/public divide

Some writers of social network studies have alluded to the importance of networks in accessing resources and adjusting to urban life especially for migrants to urban areas. Since men mostly work outside the home, they tend to rely on weak social ties they cultivate with acquaintances at work. Granovetter (1973:361) defines the strength of a tie as ‘a combination of the amount of time, the emotional intensity, the intimacy (mutual confiding), and the reciprocal services which characterize that tie’. Men in the slums normally rely on weak ties as they do not have time to cultivate close relationships with those in their community and they form friendships at work. These weak ties can serve as bridges that can facilitate access to jobs, information and resources outside the community prompting Granovetter to write about the strength of weak ties.

In their productive lives, weak ties give men an advantage since they can access resources located elsewhere and can take advantage of opportunities outside of their immediate community because of having access to a variety of information sources. For example, a 75 year old man living alone pointed out that he migrated to Nairobi and started his own timber company after his former employer had told him of this opportunity.

What happened was that I went to Kibera to deliver timber for construction to a Nubian called Sherry. After I brought that timber he told me Lungalunga is like Kibera. It is all one land and the leader was called Leakey. So I was told there is a place called Viwandani where I could sell timber and it is in Lang'ata constituency. At that time people in this place used to live in structures made of cartons.

Indeed, opportunities created by such networks formed in the workplace play a critical role in migration decisions for men. When asked about their reasons for migrating to Nairobi most men pointed to job opportunities they had heard of from acquaintances whilst women mostly cited lack of land in rural areas. Whereas men were mostly being pulled by perceived availability of jobs, women were being pushed mostly by lack of land (see Ezeh et al., 2006). However, a number of men also pointed to land scarcity in their rural homes as a factor that forced them to migrate to Nairobi.

Women in most cases prefer to work closer to home, to be able to juggle their jobs with their domestic demands, especially following their start of reproduction. This leads them to form strong ties with other women within their communities. For example, when looking for work, they often go with other women within their localities. Although women might not be able to get a wide variety of information and to access resources from areas far from their own communities, they are often well informed about what is happening in their communities. The strong ties that women formulate with others in their community afford them economic and social security when it comes to access to food and health care services. For instance, in the IFD study when asked what their parents did if there was no food in the house, often, children pointed out that fathers would simply go out to look for work whilst mothers could look for work as well as ask their neighbors for help.

However, as people become old and retire from work, strong ties within their communities give women some advantage over men (see Knodel & Ofstedal, 2003; WHO, 2001 for a discussion of social support networks). This is mostly because, in old age, people become isolated since they cannot travel as much as they used to so men can not count on their weak ties for the day to day help they may need. For example, when an older person is a care giver, taking a sick adult child to the hospital takes a lot of energy and resources, which the older person may not have. As a result, the older care giver may need assistance from other community members. If an older person lives alone, then strong social networks can improve the person's quality of life. The strength of such ties could influence access to and utilization of medical services. For instance, one elderly man who lived alone experienced a delay in accessing medical health when he fell ill. He pointed out that he had started showing tuberculosis symptoms in January 2004 and had started receiving treatment in June of the same year when he was assisted by the catholic sisters to access treatment. He did not have the resources to start treatment earlier. For older persons living alone, their network ties could impact on whether they could access services on time. The NUHDSS data indicates that for both men and women, the proportion of older people seeking health services was relatively higher for older people who lived with at least one other adult compared to those who lived alone. During interviews, some respondents pointed out that some older people get sick and died in their houses and are not discovered for several days. The main cause of death according to respondents is sometimes not the illness which can be cured but hunger and lack of medical attention.

Interviews with older female respondents were often interrupted by people coming to check on the older women but that rarely happen during interviews with older men. This shows that older women compared to older men were well integrated into their communities and have strong ties that they could mobilize as a resource if they needed assistance.

An analysis of qualitative interviews indicates that it was highly likely that an elderly sick woman would access health services much quicker than her male counterpart. Because of their domestic role as nurse to the family, women generally knew where to go to access the health services they needed. Social networks that an individual cultivate can have an impact on their health and the health statuses of others within their household. Montgomery and Hewett (2004:6) reached a similar conclusion when they noted that,

Individual women and households are connected to others in their neighbourhoods through social network ties, and along such social circuits there may flow information about how to recognize and respond to health threats, and where appropriate services can be found.

Ezeh et al. (2006:6–9) pointed out that older ‘women (34%) are more likely than men (26%) to receive support from religious organizations, or the government, probably because they are more likely to reside with younger children unlike the men’. An additional explanation to that given by Ezeh et al. (2006) could be that some men might not know of the existence of such services or might be embarrassed to utilize support from them. Most women knew not only where to get free health services when sick but also knew where to get specialized health services if they needed them. This can also impact on their health since, as mentioned earlier, one might be sick and not be able to get medical attention. For example, some women pointed out that if they were sick they could access help to get free medical attention from the catholic sisters. However, they pointed out that some people did not utilize this service because they did not know of its existence or thought wrongly that the ‘catholic sisters’ and ‘fathers’ only helped those of the catholic faith. Women were often quite conversant on which hospitals to utilize for specific ailments. For example, for children infected by HIV/AIDS, some women often reported taking their children to a children's hospital called Lea Watoto for medicines, and they would go to other organizations to look for food.

Also, because women cultivated strong social ties with other women within the community, they were more likely to get assistance from other community members compared to men. This tallies with Amuyunzu-Nyamongo and Ezeh (2005) observation that community support often comes to those who also support the community. In IDIs and FGDs, in the Aging study, it emerged that in most cases men reported that people in their community were not helpful at all, whilst most women reported having received help such as cleaning the house, fetching water, cooking and washing clothes from neighbors or friends. This covers the domestic tasks necessary for survival. Qualitative interviews show that older people who were well integrated within their communities, regardless of the illness troubling them, normally had a positive outlook on life. Ericksen and Yancey, (cited in Granovetter, 1983:212–213) asserts that, ‘strong networks seem to be linked both to economic insecurity and a lack of social services. As long as the unemployment rate is high the threat of living in poverty is real, and as long as large segments of the population find access to medical services, day care, and social welfare services problematic, we can expect to find reliance on strong networks to continue among them’. Under conditions of generalized poverty, as is the case in the two slum areas, strong ties are important as a prerequisite to good health and to accessing help when needed. As people get older, especially when they live alone, these strong ties become a necessity.

On the other hand, existing quantitative data from the NUHDSS paints a different picture from that of qualitative research regarding the issue of health and treatment. As shown in Table 4, for both Korogocho and Viwandani, more women than men reported being sick in the past two weeks prior to the 2003 NUHDSS round of updates but fewer women than men had sought medical treatment.

These statistics might not necessarily indicate more health seeking behaviour for men as compared to women but may be a result of the patterns of morbidity. For example, more women suffered from musculoskeletal illnesses which many older people might not seek treatment for, since there is an erroneous belief that the muscular and bone aches that people suffer as they grow older are not illness but a normal process of getting old. However, more men than women suffered from respiratory illnesses, gastrointestinal illness as well as illness associated with the central nervous system for which people, regardless of gender, are more likely to seek treatment for. So there might be a need to break down health seeking behaviours as well as the need for research to understand the social constructions of illness within the slum.

## Domestic work and the male ‘disadvantage’

As a result of their previous experience with house work, women were generally more self reliant and could do most of the domestic task concerned with social reproduction, such as cooking and washing (if they were of good health and not very old). On the other hand, men could not carry out these tasks as effectively as women. Even the very old women sometimes carried out such domestic tasks although a number pointed out that they could not perform these tasks anymore. One woman who could not move far because her leg was always in pain pointed out that she could still cook (provided someone brought her water) and she could still wash herself and clean the house. On the other hand, most men pointed that they rely on others such as paid help, or free help from other female relatives to meet such needs. Older men living with their wives and children were at an advantage because their wives and children would do the domestic chores. However, most of the older males who lived alone said they paid some women to do the work for them even if it meant sacrificing on their own food intake. What the following 88-year old man pointed out was typical of the responses of such older men living alone who could afford to pay for domestic services:

Res: There are women who walk around this place looking for employment. So I hire one and tell them to wash, cook and do such other things. However, when they cook beans, I can stay without cooking for even four days. I simply take a little at a time. Heat and eat it….Therefore she will return after four days and I give her dirty things to clean. Then she makes more food.

Int: When you have to pay, do you have difficulty in paying for these services?

Res: I get problems. I have to deny myself a lot of things in order to afford it. This is because if I don't pay the servant I will have problems. So I deny myself and give her. If I get ten shillings, then I give her so she can do a job worth ten shillings. If I get twenty shillings then she can do a job worth twenty shillings.

Although there are no other responses like this, where older men cut down some items which might include food intake or number of meals per day so as to be able to afford domestic help, to the extent that this happens to other older men, it might have negative nutritional impacts on their lives. Older men required domestic help for a wide range of activities such as fetching water, cooking, washing clothes and cleaning the house.

One salient factor that could expose older men to serious health risks after the death of their wives and if they lived alone is their inability to cook for themselves. Sometimes although the men could perform these tasks, they often find these activities embarrassing to do. Some respondents noted that older men with wives were much better off than older men with no wives. A 72-year old man who was sick and no longer worked said that his life was not extremely bad because:

My wife has been running around to fend for us. Like today she left very early for the market to buy vegetables to sell here and get some money for the family.

Although some older men in good health preferred to live alone because of financial constraints, older single men often thought it would be better to live with someone as that person could provide food and company.

## Isolation

One factor that exposed older people to health risks was social isolation. Older people felt isolated physically and socially since they were sometimes too old to visit their friends and other people in their age group. In most cases when asked how others in their age group were fairing, regardless of gender, older people invariably answered that they did not know since they never visited them. If they met fellow older people on the road, they could talk to each other but rarely did they visit each other. According to Montgomery and Hewett (2004) when older people say they are in good health, it is often in comparison to others of their age. However, in the sample from Viwandani and Korogocho, as a result of the physical isolation from other older people in their communities, older men often compared themselves to their former selves or to some younger men within their households or communities.

When the comparisons are consistently unfavourable, this may bring on feelings of resentment and inequity, producing stresses and anxieties that undermine mental health. There is reason to think that such mechanism can affect health more broadly. (Montgomery & Hewett, 2004:6)

Unfair comparisons can work to the detriment of the older persons' health.

## Conclusion

The division between the domestic and public sphere can impact on the health of older people as they grow older. As a result of the preoccupation of men in the public sphere in earlier life, they may have greater difficulty looking after themselves in old age. Their inability or reluctance to perform domestic chores that might be essential for their healthy living in old age stands out as a major factor affecting the health and well-being of older men. Because of neglect of these mundane domestic sphere chores, older men may sacrifice their nutritional needs and the need to live in a clean environment exposing themselves to illness and early death compared to older women. For women, this advantage may sometimes be eroded with advancing age. For instance, early in old age (say in 50s, 60s and 70s) most older women could still perform domestic chores for themselves. As they grow much older (say in the late 70s and beyond) they may begin to develop physical incapacities especially with osteo-arthritis that could erode this advantage and put them in a similar situation as older men. However, given their strong local social networks developed early in their lives, they do fare better and therefore suffer less isolation.

The social networks people cultivate during their life course could be very essential to accessing support in later years. Such support may include information on available health services as well as using these health services effectively. Social networks can also mitigate the negative effects of loneliness and alleviate feelings of alienation and isolation that can actually be detrimental to health, especially in old age. Feelings of isolation and alienation can sometimes make men indulge in risk behaviours, such as drinking excessively which itself has major health implications. For ‘older people living alone can lead to isolation and subsequently to illness and inadequate attentions to physical health’ (Brockerhoff, 2000). Thus, for the older male with no wife, nutritional needs are sometimes sacrificed impacting negatively on health.

The evidence in this paper suggests that social support groups for men might help mitigate some of the causes of ill-health and general unhappiness among men in the slums. These support groups can alleviate social isolation and provide older men with a sense of belonging and usefulness especially if they can be of assistance to other older men. The support groups can equip men with the necessary skills they need to lead a healthy and active life in old age as well as provide them with the necessary information they might need to enhance their health and well being.

Older people are also negatively affected by the increasing burdens of having to look after their orphaned grandchildren. One way to anticipate and mitigate the level of morbidity and mortality among the young adults will be to offer a comprehensive reproductive health education to equip them with skills to avoid HIV/AIDS infections. At the same time people who have been tested and found to be positive could be provided with support on healthy and positive living and free ARV treatment when needed. These would not only lengthen their lives so that they can look after their families, but also to improve their quality of life. This will also help improve the overall health of older people, especially older women as they would not be overly burdened with looking after their orphaned grand children or their increasingly sick adult children.

## Figures and Tables

**Table 1:** Composition of Focus group discussions.

Age group	Sex	Number of groups	Number of participants	Characteristics of groups
15–24	Female	2	8	Groups consisted of young people who may or may not married and who may or may not have elderly parents
	Male	2	8	
25–29	Female	2	8	Groups consisted of mainly married and most likely with elder parents
	Male	2	8	
50–59	Female	4	8	In our categorization they are elderly
	Male	4	8	
60+	Female	4	8	These are elderly people
	Male	4	8	
Service providers	Mixture of men and women	2	10	Health providers, community leaders, extension workers, NGO workers

**Table 2:** Characteristics of respondents in Individual in-depth interviews.

Characteristics	Village	Number	Men	Women
Older people dealing with recent death of an adult child	Viwandani	4	1	3
	Korogocho	4	3	1
Older people looking after orphans	Viwandani	3	1	2
	Korogocho	2	0	2
Disabled older people	Viwandani	4	2	2
	Korogocho	4	1	3
Older people living alone	Viwandani	6	3	3
	Korogocho	5	4	1

**Table 3:** Working status.

	Total population 60+		Korogocho population 60+		Viwandani population 60+	
	Women	Men	Women	Men	Women	Men
Percentage currently working	51.5	62.1	52.3	63.1	46.9	59.9
Type of work						
Petty trading	72.7	50.2	71.9	57	78.3	34.2
Formal employment	20.8	22.9	21.3	16.1	17.4	39
Informal employment	6.6	26.9	6.9	26.9	4.4	26.8

Statistics from Ezeh et al. (2006).

**Table 4:** Health status and treatment seeking behaviour among older people, Korogocho and Viwandani by gender Nairobi Demographic and Health Surveillance System, 2003.

Health status and care seeking	Total population 60+	
	Women	Men
Sick (past two weeks)	29.6	18.1
Types of illness		
Musculoskeletal	57.1	43.8
Respiratory system	15.2	22.5
Gastrointestinal system	5.7	7.5
Central nervous system	13.3	16.3
Other	8.6	10
Sought care for illness	44.8	47.5

## Footnotes

1 Mod is short for moderator, Int is short for interviewer, Res: Is short for respondent and where there is more than one respondent the respondents are given numerical numbers for identification.
